# Tumour heterogeneity revealed by unsupervised decomposition of dynamic contrast-enhanced magnetic resonance imaging is associated with underlying gene expression patterns and poor survival in breast cancer patients

**DOI:** 10.1186/s13058-019-1199-8

**Published:** 2019-10-17

**Authors:** Ming Fan, Pingping Xia, Bin Liu, Lin Zhang, Yue Wang, Xin Gao, Lihua Li

**Affiliations:** 1 Institute of Biomedical Engineering and Instrumentation, Hangzhou Dianzi University, Xiasha High Education Zone, Hangzhou, 310018 Zhejiang China; 2 Department of Electrical and Computer Engineering, Virginia Polytechnic Institute and State University, Arlington, VA 22203 USA; 3 Computational Bioscience Research Center (CBRC), Computer, Electrical and Mathematical Sciences and Engineering Division (CEMSE), King Abdullah University of Science and Technology (KAUST), Thuwal, 23955-6900 Saudi Arabia

**Keywords:** Convex analysis of mixtures, Dynamic magnetic resonance imaging, Recurrence-free survival, Gene pathway analysis, Breast cancer

## Abstract

**Background:**

Heterogeneity is a common finding within tumours. We evaluated the imaging features of tumours based on the decomposition of tumoural dynamic contrast-enhanced magnetic resonance imaging (DCE-MRI) data to identify their prognostic value for breast cancer survival and to explore their biological importance.

**Methods:**

Imaging features (*n* = 14), such as texture, histogram distribution and morphological features, were extracted to determine their associations with recurrence-free survival (RFS) in patients in the training cohort (*n* = 61) from The Cancer Imaging Archive (TCIA). The prognostic value of the features was evaluated in an independent dataset of 173 patients (i.e. the reproducibility cohort) from the TCIA I-SPY 1 TRIAL dataset. Radiogenomic analysis was performed in an additional cohort, the radiogenomic cohort (*n* = 87), using DCE-MRI from TCGA-BRCA and corresponding gene expression data from The Cancer Genome Atlas (TCGA). The MRI tumour area was decomposed by convex analysis of mixtures (CAM), resulting in 3 components that represent plasma input, fast-flow kinetics and slow-flow kinetics. The prognostic MRI features were associated with the gene expression module in which the pathway was analysed. Furthermore, a multigene signature for each prognostic imaging feature was built, and the prognostic value for RFS and overall survival (OS) was confirmed in an additional cohort from TCGA.

**Results:**

Three image features (i.e. the maximum probability from the precontrast MR series, the median value from the second postcontrast series and the overall tumour volume) were independently correlated with RFS (*p* values of 0.0018, 0.0036 and 0.0032, respectively). The maximum probability feature from the fast-flow kinetics subregion was also significantly associated with RFS and OS in the reproducibility cohort. Additionally, this feature had a high correlation with the gene expression module (*r* = 0.59), and the pathway analysis showed that Ras signalling, a breast cancer-related pathway, was significantly enriched (corrected *p* value = 0.0044). Gene signatures (*n* = 43) associated with the maximum probability feature were assessed for associations with RFS (*p* = 0.035) and OS (*p* = 0.027) in an independent dataset containing 1010 gene expression samples. Among the 43 gene signatures, Ras signalling was also significantly enriched.

**Conclusions:**

Dynamic pattern deconvolution revealed that tumour heterogeneity was associated with poor survival and cancer-related pathways in breast cancer.

## Background

Breast cancer is the most common malignancy in women. Neoadjuvant chemotherapy (NAC) is commonly used to treat patients with large and locally advanced breast tumours with the aim of decreasing tumour size, thereby minimizing micro-metastatic disease. In patients who achieved a pathologic complete response (pCR) after NAC, both the overall survival (OS) and recurrence-free survival (RFS) rates were favourable [1]. However, not all patients who receive NAC can benefit from this treatment; some patients have a poor pathological response and suffer from the toxicity and side effects associated with chemotherapy. Therefore, it is crucial to identify the prognostic factors that can be used to determine an optimal chemotherapy regimen to maximize the clinical outcome.

Dynamic contrast-enhanced magnetic resonance imaging (DCE-MRI) is a technology that has the ability to monitor tumour morphological and physiological characteristics by measuring the enhancement velocity of the contrast material in a noninvasive way. Various studies have been performed to quantitatively evaluate DCE-MRI phenotypes through radiomic/radiogenomic analyses for their association with genomic features [2–4], breast cancer subtypes [5], treatment response [6–8] and patient RFS [9]. Yamamoto et al. identified DCE-MRI features associated with early metastasis-related lncRNA radiogenomic biomarkers, which helped to elucidate genetic/molecular disease mechanisms [10]. Mazurowski et al. extracted MRI phenotypes from 48 patients and discovered their associations with luminal B subtypes of breast cancer, providing a potential noninvasive technology for determining clinical diagnostic indicators [11]. Although progress has been made, obstacles remain that impede the clinical utility of this technology.

Tumour heterogeneity not only among different tumours but also within individual tumours is common in breast cancer. A study has revealed that spatially separated regions within a single tumour exhibit distinct gene expression signatures of good and poor prognoses [12]. Regarding tumour imaging, different areas within a tumour may have varying dynamic enhancement patterns on MRI. Studies have attempted to identify tumour subregions by clustering the dynamic signals of pixels and further examining the specific dynamic patterns of imaging features to identify an association with prognosis or response to NAC [7, 13, 14] in breast cancer patients. However, due to the limited imaging resolution of DCE-MRI, each of the observed pixels may be a reflection of the pixel-wise spatially mixed partial volume effect (PVE), which is composed of multiple distinct dynamic patterns in those breast tumour areas on MRI [15]. An accurate representation of this effect on DCE-MRI is vitally important to better reveal tumour heterogeneity. To this end, previous studies have proposed identifying tumours that exhibit a unique kinetic pattern with an unsupervised method for deconvoluting a dynamic imaging series [16, 17] of tumours with heterogeneous signals using a convex analysis of mixtures (CAM) method. However, not enough literature is available to demonstrate whether the imaging phenotype inside a tumour, rather than that of the entire tumour, can augment the performance of survival prognosis in breast cancer.

The purpose of this study is to evaluate intratumoural heterogeneity based on decomposed DCE-MR images and to evaluate imaging features inside these heterogeneous regions for the determination of breast cancer prognosis. The gene signatures that are associated with the prognostic imaging features are also identified. These gene expression signatures are further examined on an independent dataset to identify their association with RFS or OS.

## Methods

### Data cohorts

The imaging dataset was collected from a publicly available dataset at The Cancer Imaging Archive (TCIA) [18], whereas the corresponding gene expression data were obtained from The Cancer Genome Atlas (TCGA) [19]. Inspired by previous work [20] showing that parenchymal features surrounding tumours were associated with breast cancer prognosis, we utilized four datasets to establish and validate the relationships between imaging phenotypes and survival data on RFS and OS. No patients overlapped among the four datasets. The demographic and clinical data for all four cohorts are presented in Table 1.

**Table 1:** Demographics of the study cohorts

Parameter	Training cohort (*n* = 61)	Reproducibility cohort (*n* = 173)	Radiogenomic cohort (*n* = 87)	TCGA cohort (*n* = 1010)
Age (years)				
Median	48 (29.7–72.4)	47.8 (26.7–68.8)	52 (29–82)	59 (26–90)
Mean ± SD	48.1 ± 9.8	47.7 ± 8.8	53.3 ± 11.3	58.9 ± 13.2
Race				
Asian	3 (5)	7 (4)	0	61 (6)
Black or African-American	3 (5)	30 (17)	4 (5)	180 (18)
White	47 (77)	135 (78)	82 (94)	675 (67)
Unknown or others	8 (13)	1 (1)	1 (1)	94 (9)
Oestrogen receptor status				
Positive	28 (46)	97 (56)	75 (86)	733 (73)
Negative	20 (33)	74 (43)	12 (14)	226 (22)
Indeterminate	0	0	0	2 (0)
Unknown	13 (21)	2 (1)	0	49 (5)
Progesterone receptor status				
Positive	22 (36)	81 (47)	69 (79)	630 (63)
Negative	26 (43)	90 (52)	18 (21)	326 (32)
Indeterminate	0	0	0	4 (0)
Unknown	13 (21)	2 (1)	0	50 (5)
Human epidermal growth factor receptor 2 status				
Positive	14 (23)	102 (59)	15 (17)	149 (15)
Negative	31 (51)	69 (40)	46 (53)	518 (51)
Equivocal	0	0	19 (22)	172 (17)
Unknown	16 (26)	2 (1)	7 (8)	171 (17)
Histological type				
Infiltrating ductal	37 (60)	N/A	75 (86)	709 (70)
Infiltrating lobular	12 (20)	N/A	10 (12)	193 (19)
Others	12 (20)	N/A	2 (2)	107 (11)
Unknown	0	N/A	0	1 (0)
Follow-up (years)				
Median	5.39 (0.28–9.84)	3.91 (0.51–6.76)	3.48 (0.37–9.40)	1.15 (0.03–19.36)
Mean ± SD	4.77 ± 2.749	3.85 ± 1.46	3.93 ± 2.22	2.37 ± 2.94
Recurrence				
Event	23 (38)	49 (28)	5 (6)	97 (10)
No event	38 (62)	124 (72)	67 (77)	674 (67)
Unknown	0	0	15 (17)	239 (23)
Death				
Event	N/A	32 (18)	1 (1)	104 (10)
No event	N/A	138 (80)	86 (99)	906 (90)
Unknown	N/A	3 (2)	0	0

Numbers in parentheses are percentages

*ER* oestrogen receptor, *PR* progesterone receptor, *HER2* human epidermal growth factor receptor 2

The first dataset (i.e. breast MRI-NACT pilot in the TCIA), which was termed the training cohort, initially included the preoperative DCE-MRI and RFS data of 64 breast cancer patients; however, no gene expression data were available for these patients. Among them, 3 patients with incomplete dynamic series were removed, resulting in 61 patients for inclusion in the training cohort.

We included an additional cohort, the reproducibility cohort, which initially included 222 breast cancer patients (from the I-SPY 1 TRIAL in the TCIA database) with available DCE-MRI and corresponding RFS and OS data [21]. We excluded 26 patients with incomplete imaging sequences, 10 with no visible tumour and 13 with low-quality images. The final dataset included samples from 173 breast cancer patients in the reproducibility cohort for analysis.

An independent dataset, termed the Radiogenomic cohort, initially included 137 patients with available DCE-MRI data from TCGA-BRCA and the corresponding gene expression data from the TCGA dataset. To reduce variation among the imaging protocols, we retained 101 patients who were evaluated with a GE 1.5-T Medical Systems imaging unit (Milwaukee, WI). After that, we excluded 1 patient who had no available gene expression data, 7 patients who had no available clinical information and 6 who had incomplete imaging data. Thereafter, the final dataset included 87 patients for analysis.

The fourth dataset, termed the TCGA cohort, included the data of 1010 patients collected from the TCGA database, all of whom had RNA sequencing data available for tumour samples along with RFS and OS data but without imaging data.

### Framework overview

**Fig. 1:**
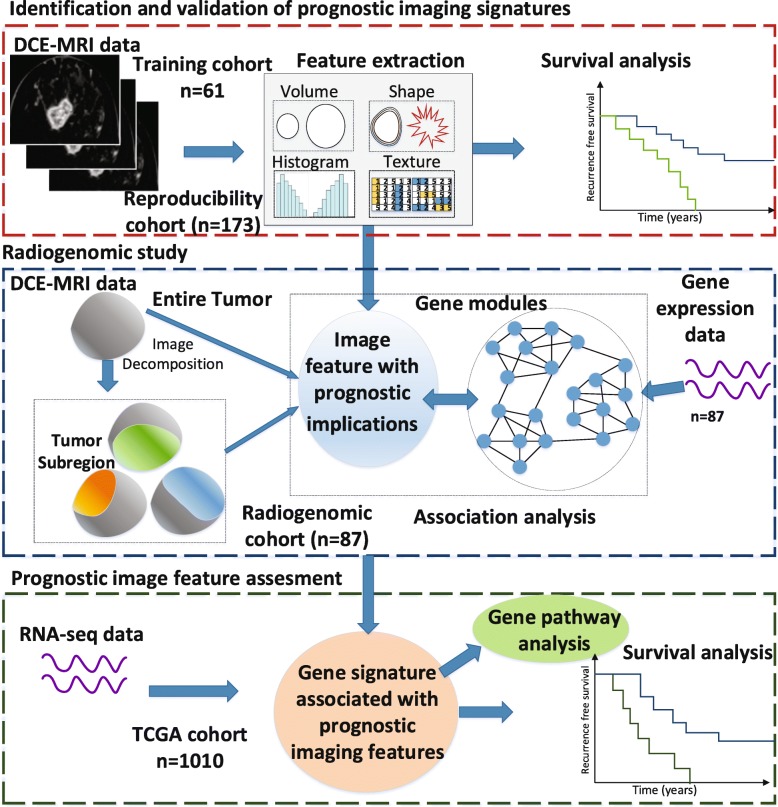
Overall framework of this study. The three modules are shown in boxes: the prognostic imaging biomarker identification and validation (red), the radiogenomic map for the gene signature (blue) and the assessment of prognostic value of gene signatures (green)

As shown in Fig. 1, the framework of this study included three modules: (i) prognostic imaging biomarker identification and validation (red); (ii) radiogenomic analysis of the association between the prognostic imaging features and gene expression for biological function analysis, followed by prognostic gene signature identification (blue); and (iii) the independent dataset for evaluating the prognostic implication of the gene signatures (green).

The prognostic features from the training cohort were first established and validated in the reproducibility cohort by associating tumour MRI features and the survival of breast cancer patients. In the Radiogenomic cohort, imaging features from the entire tumour and CAM-based tumour subregions were extracted from both the entire tumour and CAM-based tumour subregions to evaluate the association with gene expression modules. Pathway analysis was performed in the gene modules that had a high correlation with prognostic imaging features that were explored in the training cohort. A linear regression model was established to identify gene signatures that were related to prognostic imaging features. These signatures were further assessed in an independent dataset (the TCGA cohort) with available gene expression and survival data (i.e. OS and RFS). Details of these cohorts have been published elsewhere [15, 22].

### Imaging protocol

DCE-MR images collected for the training cohort were acquired using a 1.5-T scanning system (GE Healthcare, Milwaukee, WI). Breast MRI examinations were performed with patients placed in the prone position. T1-weighted, fat-suppressed MR images were acquired using the following parameters: repetition time (TR) = 8 ms, echo time (TE) = 4.2 ms, matrix = 256 × 192 × 60, flip angle = 20°, field of view = 180–220 mm, in-plane resolution = 0.7–0.9 mm and slice thickness = 2–2.4 mm. A bolus of 0.1 mmol/kg gadobutrol was intravenously injected using an MRI-compatible power injector. The early and late postcontrast images were obtained 2.5 min and 7.5 min after the contrast material injection, respectively, using standard *k*-space sampling.

For the reproducibility cohort, MRI was performed on a 1.5-T scanner using a dedicated breast radiofrequency coil. A contrast-enhanced, T1-weighted series was acquired in the sagittal orientation. A three-dimensional, fat-suppressed, gradient echo sequence was acquired with TR ≤ 20 ms, TE = 4.5 ms, flip angle ≤ 45°, field of view = 160–180 mm, minimum matrix 256 × 192, 64 slices, slice thickness ≤ 2.5 mm and in-plane spatial resolution ≤ 1 mm. The precontrast sequence was acquired, followed by early-phase and delayed-phase sequences at 2.5 min and 7.5 min after the contrast material injection, respectively.

For the Radiogenomic cohort, DCE-MRI data were collected from the TCGA-BRCA dataset, which includes data contributed by four institutions, including the Memorial Sloan Kettering Cancer Center, the Mayo Clinic, the University of Pittsburgh Medical Center and Roswell Park Cancer Institute. The imaging protocols included one precontrast image and three to five postcontrast images obtained using a T1-weighted, 3-dimensional (3D) spoiled gradient echo sequence with a gadolinium-based contrast agent. The typical in-plane resolution was from 0.53 to 0.85 mm, the typical spacing between slices was from 2 to 3 mm and the flip angle was 10°.

### DCE-MRI decomposition by CAM

After the manual annotation of the centre location of the suspicious breast tumour, image segmentation was performed on each series using a fuzzy C-means (FCM) algorithm [23]. After that, CAM was performed to decompose dynamic signals for each pixel. We defined the scan series of tumour dynamic enhancement signals for each pixel *i* as *x*(*i*), and the time-series curve in a heterogeneous tumour tissue can be modelled as the linear combination of the time-series curves *a*_*j*_(*t*) from each type of tissue, weighted by the tissue type proportions *K*_*j*_(*i*) at that pixel. Assuming that every tissue type has a similar dynamic enhancement pattern, the signal decomposition problem can be addressed using the following equation:

$$ x(i)=\left\{\sum \limits_{j=1}^J{K}_j(i){a}_j|{K}_j(i)\ge 0,\sum \limits_{j=1}^J{K}_j(i)=1,i=1,\cdots, N\right\}, $$

where *a*_*j*_ is a nonnegative vector of the time-series dynamic signal *a*_*j*_(*t*) over time, and *J* is the number of mixed tissue types reflecting distinct kinetic patterns. This method first applies affinity propagation clustering [24] of voxels into an optimal number of representative clusters, i.e. {*x*_*m*_}, and the mixture model was fitted by an expectation-maximization method. More specifically, CAM was performed to identify the tissue-specific pixel clusters spatially located at the corners of the clustered pixel time-series scatter simplex via a minimum error margin convex hull for data fitting:

$$ {\updelta}_{m,\left\{1,\cdots J\right\}\epsilon {C}_J^M}=\mathit{\min}{\left\Vert {x}_m-{\sum}_{j=1}^J{a}_j{x}_j\right\Vert}_2,{a}_j\ge 0,{\sum}_{j=1}^J{a}_j=1. $$

Thereafter, the time-series dynamic signal for each pixel was decomposed into several tissue types with certain proportions. An image pixel *i* is determined to belong to a specific tissue type if its value of proportions *K*_*j*_(*i*) is nontrivial (i.e. larger than 1e^− 2^). Therefore, a pixel was referred to as a mixture of several different tissue types if there were various nontrivial values of the tissue type proportions for this pixel. The number of underlying vascular compartments was detected using the minimum description length (MDL) of the model. In our previous studies, we performed convex analysis of mixtures (CAM) on tumour images to decompose the tumours into three compartments corresponding to plasma input, fast-flow kinetics and slow-flow kinetics. Using these criteria, most of the cases showed an optimal number of three subregions [15, 17]. To make a fair comparison, we set the number of tumour subregions to three in the current study.

### DCE-MRI feature extraction

Based on the tumour subregions generated by CAM, we extracted features inside these regions on the precontrast series, on the image subtractions between the postcontrast image series (i.e. the early postcontrast (approximately 2.5 min) and the late postcontrast (approximately 7.5 min)) and on the precontrast series, which were termed S-0, S-1 and S-2, respectively. The histogram-based features included the skewness, kurtosis and median value of the tumour images. Haralick features that measure the textural heterogeneity based on the grey-level co-occurrence matrix (GLCM) were calculated, including the energy, maximum probability and correlation. Both the histogram features and Haralick features were obtained on the image series of S-0 and S-1. The morphological features of volume and compactness were also evaluated on S-0. We omitted performing CAM on imaging data from the training cohort because most of the imaging series of these patients only had two postcontrast series, which would have resulted in inaccurately decomposed subregions. All image processing and feature extraction processes were performed in MATLAB (MathWorks, Natick, MA).

### Identification and validation of image biomarkers in breast cancer survival analysis

We evaluated the prognostic value of image features in the training cohort by individually establishing their associations with the patients’ RFS. Furthermore, a multivariate Cox regression model using all of these features was established to evaluate which features were independently associated with RFS. The prognostic value of the image features was confirmed using an additional, independent dataset using available DCE-MRI data and survival data of RFS and OS.

### Image feature function analysis by association with gene pathways

To establish relationships between the tumour image phenotype and the corresponding gene expression, we extracted the identical image features from the Radiogenomic cohort to those in the training cohort from the entire tumour and from the tumour subregions. Based on the corresponding gene expression data, gene module analysis was performed to identify a small number of representative genes that were associated with image features. We used a weighted gene co-expression network with block-wise module function to identify the gene expression modules [25]. An eigengene in each module was measured by the first principal component of the module expression profiles, which explains the maximum amount of variation in the module expression levels. Pearson correlation analysis was computed to assess the association between modules (i.e. eigengenes) and the image features. For the gene modules that showed high correlations with image features, pathway analysis was performed using Kyoto Encyclopedia of Genes and Genomes (KEGG) pathway analysis to identify the significantly enriched molecular pathways and to explore the biological importance of the imaging features.

### Radiogenomic analysis for associating gene signatures with prognostic imaging features

Inspired by the idea from previous studies that the prognostic value of the image features is evaluated by leveraging survival data in gene expression datasets [20, 26, 27], we established a radiogenomic map by identifying gene signatures associated with the prognostic imaging phenotype. To this end, gene signatures from the whole genome were identified to determine their associations with the prognostic imaging phenotype from MRI data. An elastic net was established for the association analysis, which was a regularized regression method that linearly combined the L1 and L2 penalties of the LASSO and ridge methods. Model parameters (i.e. *α* and *λ*) were selected by applying a tenfold cross-validation to reduce potential model overfitting. The tumour genes that constituted the signature were investigated using the KEGG pathway enrichment analysis to confirm the previously identified pathways that were enriched in the entire tumour or tumour subregions.

### Assessment of the prognostic value of gene signatures for image features

Gene signatures were identified by the radiogenomic link between the prognostic image features and gene expression data from the Radiogenomic cohort. We used tumour gene expression-based signatures for the image features, testing their prognostic value by assessing the associations with RFS and OS in independent cohorts from the TCGA cohort. Based on these gene signatures and the estimated parameters in the Radiogenomic cohort, a regression model was established, and the same threshold as that of the survival model in the training cohort was applied to stratify patients with different prognoses.

### Statistical analysis

The univariate and multivariate Cox proportional hazards models were both used to build survival models associated with OS and RFS. Kaplan-Meier analysis was used to estimate survival probability. We determined the optimal threshold value as the cutoff point with the smallest log-rank *p* value in the training cohort to identify prognostic imaging features. The Harrell's concordance index (c-index) and the log-rank test were used to assess the prognostic performance. The hazard ratios (HRs) with 95% confidence intervals (CIs) were assessed to compare the OS and RFS rates between the stratified groups on Kaplan-Meier plots.

To control the false discovery rate (FDR) in multiple statistical testing, the Benjamini-Hochberg method was used in the univariate survival analysis. FDR-corrected *p* values of less than 0.1 were considered to be statistically significant. The hypergeometric test was used to assess whether genes within a particular pathway were significantly overexpressed. All statistical analyses were performed in R (R Foundation for Statistical Computing, Vienna, Austria).

## Results

### Prognostic image feature identification and validation

**Fig. 2:**
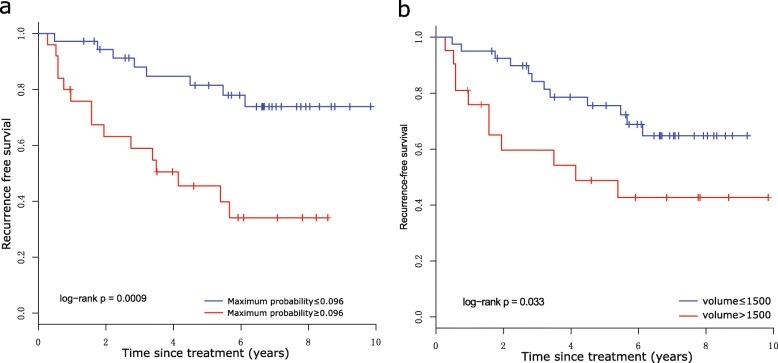
The image features of **a** maximum probability and **b** volume are used to stratify patients with different prognoses

The prognostic significance of the 14 MRI features was assessed, and the results showed that features including volume, median value, compactness, maximum probability in the precontrast series and the median value in the postcontrast series were significantly (corrected *p* values < 0.05) associated with RFS (Table 2). Among them, the feature maximum probability stratified patients with significant differences (*p* = 0.0009) in RFS, and the optimal threshold was 0.096 (Fig. 2).

**Table 2:** Image features for survival analysis

Feature	Beta	HR (95% CI)	Wald test	*p* value	Corrected *p*
Volume^†^	0.47	1.6 (1.2–2.2)	8.4	0.004	0.024
Maximum probability^†^	0.46	1.6 (1.1–2.2)	7.9	0.005	0.024
Median^‡^	0.44	1.6 (1.1–2.1)	7.6	0.006	0.024
Median^†^	0.44	1.6 (1.1–2.1)	7.3	0.007	0.024
Compactness^†^	0.43	1.5 (1.1–2.2)	6.4	0.011	0.031
Energy^†^	0.34	1.4 (1.0–1.9)	5.2	0.023	0.054
Skewness^†^	− 0.56	0.57 (0.3–1.1)	2.9	0.087	0.170
Correlation^†^	0.25	1.3 (0.85–1.9)	1.4	0.230	0.400
Correlation^‡^	0.24	1.3 (0.79–2.0)	1.0	0.320	0.500
Maximum probability^‡^	− 0.11	0.9 (0.57–1.4)	0.20	0.650	0.820
Kurtosis^‡^	0.086	1.1 (0.72–1.7)	0.16	0.690	0.820
Energy^‡^	0.073	1.1 (0.75–1.6)	0.15	0.700	0.820
Kurtosis^†^	0.047	1.0 (0.76–1.4)	0.08	0.770	0.830
Skewness^‡^	− 0.014	0.99 (0.67–1.4)	0	0.940	0.940

*p* values were adjusted by the Benjamini-Hochberg method

*HR* hazard ratio, *CI* confidence intervals

^†^Precontrast series (S-0)

^‡^Subtraction between the early postcontrast and precontrast series (S-1)

After removing features with high similarity to each other (i.e. Pearson correlation coefficient between two image features greater than 0.7), a multivariate Cox regression analysis was performed using seven features. More specifically, the image features of skewness, correlation and maximum probability in S-0, and kurtosis, skewness, median value and maximum probability in the postcontrast series were used and included in the multivariate Cox regression model. The results showed that the maximum probability obtained in the S-0 was the most significant feature that was independently correlated with RFS (*p* = 0.0018). In addition, the image features of the median value of S-1 and tumour volume were independently associated with RFS with *p* values of 0.0036 and 0.0032, respectively.

We performed a survival analysis using the same 14 MRI features in the reproducibility cohort to analyse any associations with RFS and OS (Table 3). Image features of volume, maximum probability and compactness were significantly associated with both RFS and OS (corrected *p* values < 0.05), which were also tested for significant association with RFS in the training cohort. Additionally, the feature of energy showed a significant association with RFS and OS with corrected *p* values of 0.042 and 0.09, respectively. As a comparison, this feature was significantly correlated with RFS, with a *p* value of 0.023, but was not significant after FDR correction (*p* = 0.054) in the training set of 61 patients.

**Table 3:** Image features for survival analysis in the reproducibility cohort

Feature	Recurrence-free survival			Overall survival		
	HR (95% CI)	*p*	FDR	HR (95% CI)	*p*	FDR
*Volume* ^†^	1.5 (1.2–1.8)	< 10^−3^	0.003	1.6 (1.3–2)	< 10^−4^	< 10^−3^
*Maximum probability* ^†^	1.3 (1.1–1.6)	0.012	0.042	1.4 (1.1–1.7)	0.001	0.006
Median^‡^	0.98 (0.73–1.3)	0.890	0.890	0.89 (0.56–1.4)	0.630	0.969
Median^†^	1 (0.78–1.4)	0.840	0.890	0.97 (0.67–1.4)	0.890	0.969
*Compactness* ^†^	1.5 (1.2–1.9)	0.002	0.011	1.7 (1.3–2.2)	< 10^−4^	< 10^−3^
*Energy* ^†^	1.3 (1.1–1.5)	0.012	0.042	1.3 (1.1–1.6)	0.003	0.009
Skewness^†^	1.1 (0.81–1.4)	0.580	0.677	1.3 (0.92–1.8)	0.150	0.300
Correlation^†^	1.2 (0.87–1.6)	0.290	0.543	1.1 (0.75–1.5)	0.690	0.969
Correlation^‡^	1.2 (0.89–1.7)	0.210	0.490	1.4 (0.91–2)	0.130	0.300
Maximum probability^‡^	0.9 (0.65–1.2)	0.520	0.662	1 (0.71–1.4)	0.980	0.980
Kurtosis^‡^	0.87 (0.59–1.3)	0.490	0.662	0.98 (0.67–1.4)	0.900	0.969
Energy^‡^	0.84 (0.57–1.2)	0.380	0.591	0.95 (0.65–1.4)	0.780	0.969
Kurtosis^†^	1.2 (1–1.5)	0.031	0.087	1.3 (1.1–1.5)	0.010	0.028
Skewness^‡^	0.84 (0.6–1.2)	0.310	0.543	1 (0.73–1.4)	0.890	0.969

*p* values were adjusted by the Benjamini-Hochberg method

The image features that are significantly associated with RFS and OS are shown in italics

*HR* hazard ratio, *CI* confidence intervals

^†^Precontrast series (S-0)

^‡^Subtraction between the early postcontrast and precontrast series (S-1)

### Association between gene modules and prognostic image features in tumours and tumour subregions

Features that were identified to have prognostic implications were further examined by evaluating the associations with the gene modules in the Radiogenomic cohort (*n* = 87) with the corresponding DCE-MRI and gene expression data. We removed genes expressed in only 20% of the patients and those with no expression values (*n* = 3759). We then deleted genes with low variance of expression across patients, and ultimately, the top 5000 genes with the largest variance were retained in the dataset. For the network construction, a pairwise correlation matrix was computed, and then an adjacency matrix was calculated by raising the correlation matrix to the power of five [25]. To obtain meaningful and distinct modules, we set the minimum module size to 60 genes and the minimum height for merging modules to 0.25. After that, we obtained 16 gene modulates. Detailed information of the top 5 significantly enriched molecular pathways in the 16 gene modules is shown in Additional file 1: Table S1.

We first examined the correlation between image features from the entire tumour and co-expressed gene modulates, and the results showed that three features had a high correlation with the gene modules (Pearson correlation coefficient > 0.5). Among them, only tumour volume, which was also identified to have prognostic implications in the training cohort, remained relatively high related to the gene modulates (Table 4).

**Table 4:** List of image features in the entire tumour and intratumoural subregions and the correlations with gene expression modules

Type	Module	Feature
Entire tumour		
Entire tumour	Tan (*n* = 158)	Kurtosis^†^ (*r* = 0.67)
	Magenta (*n* = 198)	**Volume**^†^ **(*r*** **= 0.6)**, compactness^†^ (*r* = 0.52)
Tumour subregions		
Plasma input	Tan (*n* = 158)	Kurtosis^†^ (*r* = 0.7); volume^†^ (*r* = 0.76); energy^†^ (*r* = 0.55)
	Magenta (*n* = 198)	Compactness^†^ (*r* = 0.54)
Fast-flow kinetics	Tan (*n* = 158)	Kurtosis^†^ (*r* = 0.68); energy^†^ (*r* = 0.56); **Maximum probability**^†^ **(*r*** **= 0.59)**
	Green yellow (*n* = 166)	Energy^‡^ (*r* = 0.58); maximum probability^‡^ (*r* = 0.52)
Slow-flow kinetics	Tan (*n* = 158)	Kurtosis^†^ (*r* = 0.55); energy^†^ (*r* = 0.6); **Maximum probability**^†^ **(*r*** **= 0.53)**
	Magenta (*n* = 198)	Compactness^†^ (*r* = 0.59)

Only image features that have a high correlation (*r* > 0.5) with the gene module are shown. The image features with prognostic implications are in bold

^†^Precontrast series (S-0)

^‡^Subtraction between the early postcontrast and precontrast series (S-1)

**Fig. 3:**
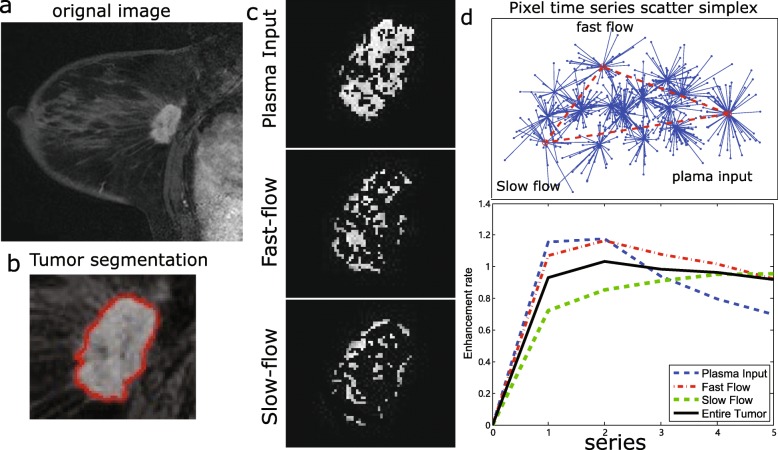
Example of CAM applied to **a** a breast image. **b** Segmented tumour image. **c** The tumour is decomposed into three regions, and images of the associated regions represent plasma input, fast-flow kinetics and slow-flow kinetics. **d** Image pixels are grouped into clusters using an affinity propagation clustering method. The clusters represented by the vertices are identified by CAM. **e** Dynamic enhancement curves for the tumour and the three tumour subregions representing tissue-specific compartments, in which the blue, red and green colours represent the plasma input, fast-flow kinetics and slow-flow kinetics, respectively

We also performed the same association analysis between gene expression modules and image phenotypes using features from the tumour subregions (Table 4). Based on the CAM analysis of breast MR images, tumours were decomposed into three compartments (Fig. 3a–c). Among all dynamic curves, regions representing plasma input showed a kinetic pattern of rapid wash-in and fast wash-out (Fig. 3d). The kinetics of the fast-flow subregion showed a slightly higher wash-in rate than that of the whole tumour, while the kinetics of the slow-flow subregion had the lowest wash-in rate and the highest wash-out rate.

**Fig. 4:**
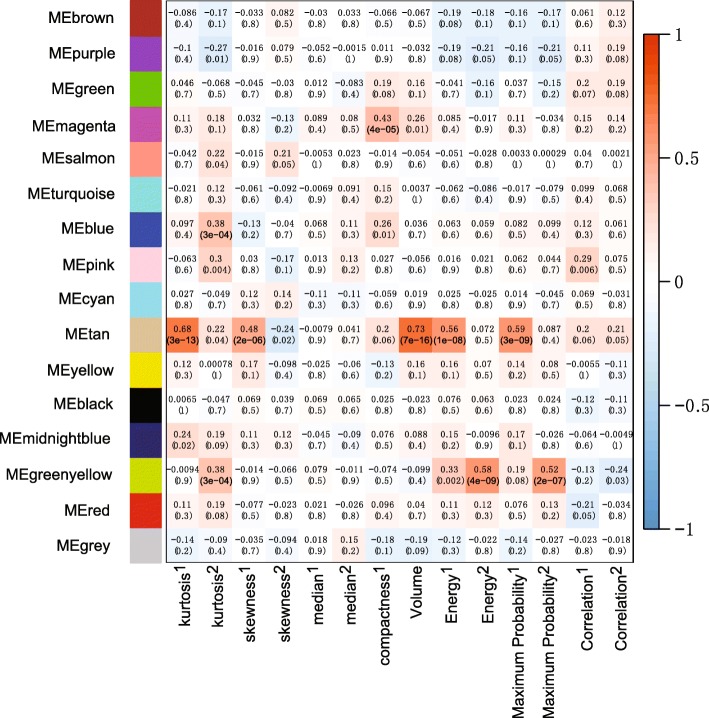
Image features from the fast-flow kinetics subregion are correlated with the gene modules

Specifically, the correlation between image features and fast-flow kinetics-related tumour subregions is shown in Fig. 4. Among them, the prognostic features of maximum probability in the tumour subregions showed an association with the gene module/eigengene (labelled tan, *n* = 158). Additionally, the features in regions with fast-flow kinetics had a higher correlation with the gene expression module than that of the features from regions related to slow-flow kinetics. The correlation between image features and the other subregions, i.e. the plasma input and slow-flow kinetics regions, is shown in Additional file 2: Figure S1 and Additional file 3: Figure S2, respectively.

**Fig. 5:**
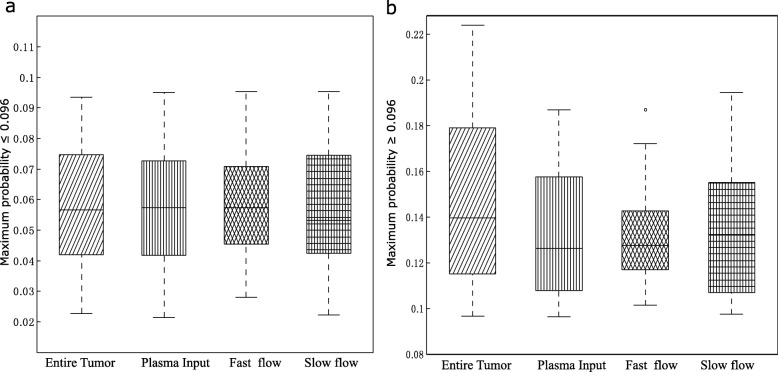
Distribution of the maximum probability feature in the entire tumour and tumour subregions in groups with **a** low and **b** high risk

We examined the distributions of the image features from the entire tumour and from the decomposed tumour subregions. In low-risk patients, the maximum probability features obtained from the tumour subregions showed a lower level of variance and a lower interquartile range than those of features based on the entire tumour, and this trend of decreased variance in the subregions compared with that in the entire tumour was more obvious for high-risk patients (Fig. 5). In other words, features from the tumour subregions had more concrete values, which may be explained by the fact that the subregions have homogeneous dynamic patterns alleviating noise information induced by tumour heterogeneity.

### Biological annotation for the modules that were associated with prognostic image features

A further KEGG enrichment analysis was performed on the gene module that was associated with prognostic image features (tan, *n* = 158) using KOBAS 3.0 (Table 5). The complete list of 158 genes with biological annotations [28] is shown in Additional file 1: Table S2. Ten pathways were significantly enriched with corrected *p* values < 0.1. Among these, the Ras signalling pathway, a tumour growth-, proliferation- and cell survival-related pathway [29, 30], was mostly enriched (*p* = 0.0044). Additionally, two significantly enriched pathways of apoptosis (*p* = 0.0165) and microRNAs in cancer (*p* = 0.0343) have been reported to be associated with breast cancer [31, 32].

**Table 5:** Pathway analysis for the tan module

Category	*p* value	Corrected *p* value
Ras signalling pathway	< 10^−4^	0.0044
Hedgehog signalling pathway	< 10^−4^	0.0044
Apoptosis	0.0003	0.0165
PI3K-Akt signalling pathway	0.0005	0.0215
Longevity regulating pathway	0.0006	0.0219
Notch signalling pathway	0.0011	0.0317
MicroRNAs in cancer	0.0014	0.0343
Spliceosome	0.0022	0.0487
Alzheimer’s disease	0.0049	0.0833
Fatty acid elongation	0.0051	0.0833

*p* values were adjusted by the Benjamini-Hochberg method

### Radiogenomic analysis identified gene signatures for prognostic image features

In addition to the biological annotation for gene modules, we built a radiogenomic map to determine gene signatures by associating gene modules with prognostic image feature indicators. We selected 100 genes that were mostly correlated with the image features and then fed them into an elastic net regression model to regress the maximum probability feature. The parameters were selected through cross-validation with *α* and *λ* values of 0.2 and 0.0037, respectively. This model selected a subset of 38 gene signatures to estimate the tumour volume feature using elastic net (*R*^2^ = 0.8159) with parameters of *α* and *λ* of 0.65 and 0.156, respectively. Additionally, we identified 43 gene signatures for regressing the image maximum probability feature from the fast-flow kinetics subregion, using elastic net with an *R*^2^ of 0.8073. Finally, the regression model (*R*^2^ = 0.8969, *α* = 0.800 and *λ* = 0.0005) with 57 gene signatures was built for predicting the maximum probability feature in the slow-flow kinetics-associated tumour subregion.

We further examined the biological functions of these gene signatures related to the maximum probability feature, and the results of the KEGG pathway analyses are shown in Table 6. After controlling for FDR, we obtained 10 enriched pathways (*p* < 0.1). The complete list of these 43 genes is shown in Additional file 1: Table S2. However, the pathway analysis for the tumour gene signatures related to the tumour volume (Additional file 1: Table S3) or maximum probability features from the slow-flow kinetics-associated tumour subregion (Additional file 1: S4) showed no significantly (corrected *p* values > 0.05) enriched pathways.

**Table 6:** Pathway analysis of 43 identified genes in the regression model

Category	*p* value	Corrected *p* value
Systemic lupus erythaematosus	0.0094	0.0779
Transcriptional misregulation in cancer	0.0159	0.0779
Glycosaminoglycan biosynthesis-keratan sulfate	0.0168	0.0779
Rap1 signalling pathway	0.0213	0.0779
Regulation of actin cytoskeleton	0.0221	0.0779
Ras signalling pathway	0.0246	0.0779
Bile secretion	0.0733	0.1740
Melanoma	0.0733	0.1740
Taste transduction	0.0850	0.1793
Ribosome	0.0137	0.2598

*p* values were adjusted by the Benjamini-Hochberg method

### Assessment of prognostic gene signatures in an independent dataset

We included 906 patients who had available OS data and 771 patients with available gene expression and survival data in the TCGA cohort. The identical gene signatures and parameters included in the previous regression model that was trained on the Radiogenomic cohort were applied using the elastic net model to regress the prognostic image features, for which both RFS and OS were associated. We evaluated the prognostic value of image features extracted from the entire tumour and from the tumour subregions by stratifying patients with different survival outcomes.

**Fig. 6:**
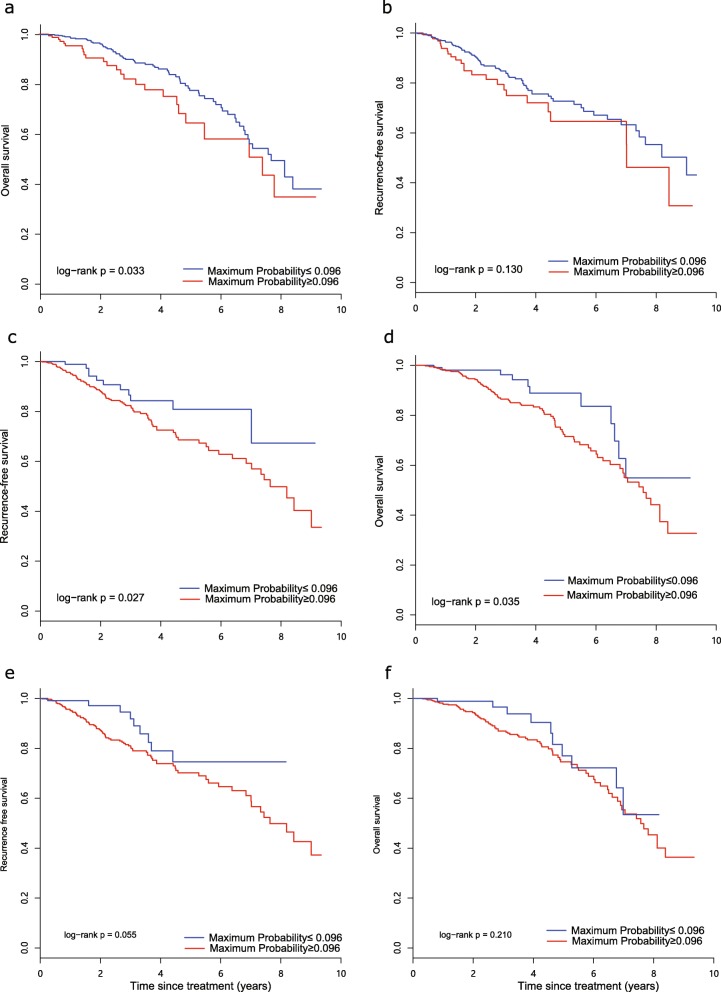
Kaplan-Meier curves of RFS and OS with maximum probability, respectively. **a**, **b** The entire tumour. **c**, **d** Fast-flow kinetics tumour subregions. **e**, **f** Slow-flow kinetics tumour subregions

Regarding the image features derived from the entire tumour, the tumour volume feature identified by gene signatures did not show a significant association with either RFS or OS, with *p* values of 0.190 and 0.200, respectively. The maximum probability feature identified by gene signatures showed a significant association with only OS (*p* = 0.033), whereas no significant association was found with RFS (*p* = 0.130) (Fig. 6a, b).

For image features extracted from tumour subregions, the prognostic value of the maximum probability was assessed, and significant results were observed for the fast-flow kinetics-related subregion based on a regression model using 43 gene signatures, which significantly stratified patients (threshold = 0.096) into 2 groups in terms of RFS and OS, with *p* values of 0.027 and 0.035, respectively (Fig. 6c, d). This feature from the slow-flow kinetics-related region showed no significant association (*p* = 0.055) with either RFS or OS (*p* = 0.210) (Fig. 6e, f). The findings indicate that the tumour subregion-based regression model exhibited better performance than that of the model based on the entire tumour.

## Discussion

This study explored the prognostic tumour features from DCE-MRI to stratify patients into groups with different survival rates. Gene expression signatures were identified by establishing their correlations with the prognostic image features. The functional information of these features, both based on the entire tumour and CAM-generated subregions, was further investigated by evaluating their associations with gene expression modules, in which breast cancer-related pathways were identified. The prognostic value of these gene signatures was confirmed in an independent dataset, which indicated significant associations between the gene signatures and patient survival. The results demonstrated that imaging features derived from tumour subregions had more prognostic value than those derived from the entire tumour did.

Previous studies [33] have associated image phenotypes with gene expression, early metastasis and long noncoding RNA expression [10]. Zhu et al. examined the relationship between multilayer molecular data from the TCGA dataset and paired DCE-MRI data from the TCIA features, including transcriptional activities of pathways, microRNA expression, protein expression, somatic mutations and gene copy number variations of all genetic pathways [34]. A related study identified tumour DCE-MRI features in the parenchymal tissue surrounding breast tumours to be associated with survival and gene pathways [20]. Our radiogenomic strategy evaluated the prognostic value of the image features by leveraging gene expression data from public gene expression datasets, which has been previously performed by several studies on nonsmall cell lung cancer (NSCLC) [26, 27] and breast cancer [20]. Our study builds upon previous work and adds to the exploration of how imaging features derived from spatially distinct tumour areas by using CAM can potentially provide useful information for breast cancer prognosis. Different from the other studies [14, 35] that used texture features to reflect the extent of heterogeneity in the entire tumour, our feature analysis was conducted in tumour subregions that exhibited specific dynamic patterns.

We identified that large tumour volume was associated with poor RFS in the training and the reproducibility cohorts, which is consistent with the findings of a previous study [36]. However, this feature regressed by gene signatures did not show a significant association with either RFS or OS in the TCGA cohort. We identified the tumour morphological feature of compactness, and its high value is associated with poor RFS. This finding is partly consistent with that of a previous study, which shows that a low value of compactness is also significantly associated with the luminal A subtype of breast cancer, which has a favourable rate of survival [5]. On the other hand, we observed that a higher value of prognostic-related maximum probability, which measures the most frequently appearing value of each pixel relationship in the GLCM, was correlated with worse survival than a lower value was. The prognostic value of this feature was assessed in the pathway analysis, which showed cancer-related biological functions. This feature, which is derived from regions with fast-flow kinetics rather than from the entire tumour, showed significant associations with both RFS and OS. One of the main causes of treatment failure (i.e. poor survival) is locoregional recurrence within a specific tumour region; this conclusion may be explained by the fact that the CAM-based decomposing method can separate spatially mixed regions caused by tumour heterogeneity and hence enhance the prognostic performance of this feature.

Notably, this texture feature derived from the precontrast image series showed better prognostic performance than that of the same feature derived from the postcontrast image series. Similar findings regarding imaging features associated with clinical biomarkers on DCE-MRI precontrast series are presented elsewhere [13, 37], findings which are partly consistent with our results. Texture features, which cannot be accurately or reliably evaluated using a visual or subjective method, may be used as candidate biomarkers that are associated with the biological characteristics of tumours.

We performed pathway analysis on both gene modules and gene signatures that were related to prognostic image features. For both analyses, we identified the same Ras signalling pathway as being significantly enriched (corrected *p* < 0.1) in both of these gene sets. This pathway, which is a key regulator of tumour growth, metastasis [38] and malignant transformation and is responsible for cell proliferation and survival [29, 30], is aberrant in most human tumours. The proteins that Ras encodes have been considered drug targets exhibiting anti-oncogenic effects in many cancer cell lines [39–41].

Despite some significant findings, several limitations should be addressed. First, our patient sample size was relatively small because only a limited number of breast MR images were available in the TCGA and TCIA databases. Further external studies should be performed to confirm the prognostic value of image features in our study. Second, DCE-MRI data were acquired from a multi-institution cohort with varied imaging parameters, introducing diversity among the images. Third, although we observed the same Ras signalling pathway that was significantly enriched (corrected *p* < 0.1) in two gene sets, the agreement was low. This result may be partly explained by the heterogeneity of the gene signature data identified by the module analysis versus that identified by the regression model. Fourth, decomposition accuracy relies on the number of image series, and this method is difficult to perform in DCE-MR image series with little time-series data, i.e. less than three postcontrast series (e.g. DCE-MRI data in the training cohort). Therefore, we did not perform CAM in the training or the reproducibility datasets, which only had one or two postcontrast series.

We performed the radiogenomic study on the entire tumour and intratumoural subregions based on the hypothesis that some specific regions are biologically more aggressive than the other regions, and subregion analysis may be more useful compared with the entire tumour in discriminating patients with different survival and in associating with aberrant gene expression. This may partly explain why the imaging features from the entire tumour shown to be prognostic did not show the same radiogenomic associations with gene expression modules as the subregion-specific features on the Radiogenomic cohort. On the other hand, although we have identified prognostic image features on the entire tumour in the first experiments, this may not directly hold for these features within the CAM generated tumour subregions. Future study is needed to confirm this study by directly validating the results on large breast cancer cohorts based on sufficient temporal resolution of DCE-MRI and the corresponding survival data to verify whether subregion analysis augment the prognostic value of radiomics in tumour. Despite these limitations, the TCGA dataset provided a unique opportunity to examine the radiogenomic associations between breast MRI and biological function and survival in breast cancer.

## Conclusion

In conclusion, intratumoural decomposition identified fast-flow kinetics tumour subregions in which DCE-MR image features were used as biomarkers for stratifying patients based on different survival rates. The prognostic image features were associated with a breast cancer-related pathway. Further work is needed before these quantitative MRI parameters can be used to facilitate the noninvasive assessment of breast cancer characteristics in clinical practice.

## Supplementary information

**Additional file 1:** **Table S1.** Top five enriched pathways of 16 gene modules. **Table S2.** Biological annotation of the 158 genes from the gene module (Tan) and the 43 genes in the signature for regressing the maximum probability feature from the fast-flow kinetics-related tumour subregion. **Table S3.** Pathway analysis of 38 identified genes in the signature for regressing the tumour volume feature. **Table S4.** Pathway analysis of 57 identified genes in the signature for regressing the maximum probability feature from the slow-flow kinetics-associated tumour subregion.

**Additional file 2:** **Figure S1.** Image features from the plasma input subregion correlated with gene modules.

**Additional file 3:** **Figure S2.** Image features from the slow-flow kinetics subregion correlated with gene modules.

## Data Availability

The datasets analysed during the current study are available from The Cancer Imaging Archive (TCIA) http://www.cancerimagingarchive.net. The gene expression data of the TCGA breast cancer cohort are available from the Genomic Data Commons https://gdc.cancer.gov/. The pathway analysis was performed using the Kyoto Encyclopedia of Genes and Genomes (KEGG) pathway in https://www.kegg.jp/.

## Abbreviations

CAM: Convex analysis of mixtures
c-index: Concordance index
CIs: Confidence intervals
DCE-MRI: Dynamic contrast-enhanced magnetic resonance imaging
FDR: False discovery rate
GLCM: Grey-level co-occurrence matrix
HR: Hazard ratio
KEGG: Kyoto Encyclopedia of Genes and Genomes
NAC: Neoadjuvant chemotherapy
OS: Overall survival
pCR: Pathologic complete response
PVE: Partial volume effect
RFS: Recurrence-free survival
TCGA: The Cancer Genome Atlas
