# Supplementary material for: Tumour heterogeneity revealed by unsupervised decomposition of dynamic contrast-enhanced magnetic resonance imaging is associated with underlying gene expression patterns and poor survival in breast cancer patients
Source: Breast Cancer Res. 2019 Oct 17;21:112. doi: 10.1186/s13058-019-1199-8 (PMC6798414; doi:10.1186/s13058-019-1199-8)
Supplement: Supplementary file 1 — Additional file 1: Table S1. Top five enriched pathways of 16 gene modules. Table S2. Biological annotation of the 158 genes from the gene module (Tan) and the 43 genes in the signature for regressing the maximum probability feature from the fast-flow kinetics-related tumour subregion. Table S3. Pathway analysis of 38 identified genes in the signature for regressing the tumour volume feature. Table S4. Pathway analysis of 57 identified genes in the signature for regressing the maximum probability feature from the slow-flow kinetics-associated tumour subregion. [file 13058_2019_1199_MOESM1_ESM.docx]

**Additional Tables**

Table S1. Top five enriched pathway of 16 gene modules

| **Module 1: Black (n=227)** | | |
| --- | --- | --- |
| Category | p-Value | Corrected P |
| Metabolic pathways | 3.509E-09 | 5.404E-07 |
| Protein processing in endoplasmic reticulum | 6.829E-06 | 0.0005258 |
| Huntington's disease | 0.0001444 | 0.007411 |
| Glutathione metabolism | 0.0002803 | 0.01079 |
| Lysosome | 0.0007984 | 0.02459 |
| **Module 2: Blue (n=458)** | | |
| Metabolic pathways | 8.38E-19 | 1.9776E-16 |
| Cell cycle | 2.656E-17 | 3.1341E-15 |
| Glycolysis / Gluconeogenesis | 4.477E-13 | 3.5222E-11 |
| Carbon metabolism | 1.962E-12 | 1.1577E-10 |
| Proteasome | 2.763E-11 | 1.3040E-09 |
| **Module 3: Brown(n=404)** | | |
| ECM-receptor interaction | 5.128E-19 | 1.021E-16 |
| Focal adhesion | 1.113E-18 | 1.108E-16 |
| PI3K-Akt signaling pathway | 1.049E-14 | 6.957E-13 |
| Proteoglycans in cancer | 1.3E-10 | 6.468E-09 |
| Protein digestion and absorption | 5.161E-10 | 2.054E-08 |
| **Module 4: Cyan (n=141)** | | |
| RNA transport | 1.292E-12 | 1.912E-10 |
| NF-kappa B signaling pathway | 9.93E-07 | 7.348E-05 |
| Metabolic pathways | 3.243E-06 | 0.00016 |
| Spliceosome | 9.794E-06 | 0.0003624 |
| Glyoxylate and dicarboxylate metabolism | 2.286E-05 | 0.0006766 |
| **Module 5: Green (n=355)** | | |
| Ribosome | 3.348E-92 | 5.691E-90 |
| Oxidative phosphorylation | 3.023E-32 | 2.569E-30 |
| Parkinson's disease | 7.439E-25 | 4.216E-23 |
| Alzheimer's disease | 3.412E-23 | 1.45E-21 |
| Huntington's disease | 8.087E-22 | 2.75E-20 |
| **Module 6: Grey (n=1026)** | | |
| Metabolic pathways | 1.13E-20 | 3.13E-18 |
| Lysosome | 2.38E-12 | 3.29E-10 |
| Regulation of actin cytoskeleton | 5.46E-10 | 5.04E-08 |
| Proteoglycans in cancer | 9.97E-10 | 6.9E-08 |
| Alcoholism | 1.87E-09 | 1.04E-07 |
| **Module 7: Midnightblue (n=95)** | | |
| Bladder cancer | 0.0001619 | 0.024765 |
| Alzheimer's disease | 0.0007855 | 0.03490 |
| TGF-beta signaling pathway | 0.0012029 | 0.03490 |
| ErbB signaling pathway | 0.0013693 | 0.03490 |
| Prostate cancer | 0.001413 | 0.03490 |
| **Module 8: Purple (n=173)** | | |
| Complement and coagulation cascades | 4.605E-09 | 7.183E-07 |
| AGE-RAGE signaling pathway in diabetic complications | 2.761E-08 | 1.64E-06 |
| HTLV-I infection | 3.153E-08 | 1.64E-06 |
| PPAR signaling pathway | 5.652E-08 | 2.204E-06 |
| Osteoclast differentiation | 2.676E-06 | 8.348E-05 |
| **Module 9: Red (n=231)** | | |
| Metabolic pathways | 2.989E-10 | 5.679E-08 |
| Ribosome | 2.188E-06 | 0.0002078 |
| Proteasome | 9.154E-06 | 0.0005798 |
| N-Glycan biosynthesis | 1.483E-05 | 0.0007046 |
| Purine metabolism | 9.534E-05 | 0.0036228 |
| **Module 10: Salmon (n=151)** | | |
| Protein processing in endoplasmic reticulum | 5.216E-05 | 0.008189 |
| Dopaminergic synapse | 0.0001694 | 0.013295 |
| cGMP-PKG signaling pathway | 0.0005168 | 0.02162 |
| Endocytosis | 0.0005508 | 0.02162 |
| Insulin resistance | 0.0009207 | 0.02742 |
| **Module 11: Turquoise(n=607)** | | |
| Staphylococcus aureus infection | 4.957E-26 | 1.264E-23 |
| Herpes simplex infection | 2.42E-25 | 3.085E-23 |
| Phagosome | 2.327E-24 | 1.978E-22 |
| Antigen processing and presentation | 2.76E-23 | 1.759E-21 |
| Viral myocarditis | 1.07E-21 | 5.457E-20 |
| **Module 12: Yellow(n=402)** | | |
| Metabolic pathways | 3.111E-08 | 6.472E-06 |
| mRNA surveillance pathway | 7.701E-06 | 0.0008009 |
| RNA transport | 1.642E-05 | 0.001139 |
| Viral carcinogenesis | 0.0003277 | 0.01677 |
| Influenza A | 0.0005602 | 0.01677 |
| **Module 13: Tan(n=158)** | | |
| Ras signaling pathway | 4.319E-05 | 0.004386 |
| Hedgehog signaling pathway | 4.984E-05 | 0.004386 |
| Apoptosis | 0.0002825 | 0.01657 |
| PI3K-Akt signaling pathway | 0.0004888 | 0.02151 |
| Longevity regulating pathway | 0.0006242 | 0.02197 |
| **Module 14: Greenyellow n=166)** | | |
| Protein processing in endoplasmic reticulum | 5.697E-08 | 8.887E-06 |
| Endocytosis | 2.229E-07 | 1.739E-05 |
| Estrogen signaling pathway | 5.403E-06 | 0.000281 |
| Adherens junction | 2.056E-05 | 0.0008018 |
| Spliceosome | 2.794E-05 | 0.0008518 |
| **Module 15: Magenta(n=198)** | | |
| Metabolic pathways | 8.486E-12 | 1.23E-09 |
| Pyruvate metabolism | 2.954E-06 | 0.0002141 |
| Citrate cycle (TCA cycle) | 2.414E-05 | 0.001084 |
| Carbon metabolism | 2.991E-05 | 0.001084 |
| Tryptophan metabolism | 6.798E-05 | 0.001972 |
| **Module 16: Pink(n=208)** | | |
| RNA transport | 1.292E-12 | 1.912E-10 |
| NF-kappa B signaling pathway | 9.93E-07 | 7.348E-05 |
| Metabolic pathways | 3.243E-06 | 0.00016 |
| Spliceosome | 9.794E-06 | 0.0003624 |
| Glyoxylate and dicarboxylate metabolism | 2.286E-05 | 0.0006766 |

Table S2. Biologic annotation for the 158 genes from the gene module (Tan) and the 43 genes in the signature for regressing the maximum probability feature from fast-flow related tumour subregion

| Hallmark name | Process category | 158 Genes | 43 Genes |
| --- | --- | --- | --- |
| 1 Apical junction | cellular component | EVL, GAMT, SHC1, CRB3 | N/A |
| 2 Apical surface | cellular component | GATA3 | N/A |
| 3 Peroxisome | cellular component | pEX11B | N/A |
| 4 Adipogenesis | development | GADD45A | N/A |
| 5 Angiogenesis | development | N/A | N/A |
| 6 Epithelial mesenchymal transition | development | AREG, GADD45A | pTHLH |
| 7 Myogenesis | development | N/A | N/A |
| 8 Spermatogenesis | development | N/A | N/A |
| 9 Pancreatic beta cell | development | N/A | N/A |
| 10 DNA repair | DNA damage | N/A | N/A |
| 11 UV response down | DNA damage | NFKB1 | N/A |
| 12 UV response up | DNA damage | NAT1, RET, pRpF3, pRKACA | N/A |
| 13 Allograft rejection | immune | N/A | ELANE |
| 14 Coagulation | immune | N/A | N/A |
| 15 Complement | immune | GATA3, CD46 | N/A |
| 16 Interferon alpha response | immune | MOV10 | N/A |
| 17 Interferon gamma response | immune | NFKB1 | N/A |
| 18 Il6 JAK STAT3 signaling | immune | N/A | N/A |
| 19 Inflammatory response | immune | NFKB1 | N/A |
| 20 Bile acid metabolism | metabolic | N/A | N/A |
| 21 Cholesterol homeostasis | metabolic | ERRFI1 | N/A |
| 22 Fatty acid metabolism | metabolic | MIF, HADHB | N/A |
| 23 Glycolysis | metabolic | MIF, CASp6 | CHST1 |
| 24 Heme metabolism | metabolic | BNIp3L, BTRC | N/A |
| 25 Oxidative phosphorylation | metabolic | NDUFS2, HADHA, HADHB | N/A |
| 26 Xenobiotic metabolism | metabolic | CASp6 | N/A |
| 27 Apoptosis | pathway | MCL1, BNIp3L, GADD45A, CASp6 | N/A |
| 28 Hypoxia | pathway | EFNA1, MIF, BNIp3L, ERRFI1, BCL2, CASp6 | N/A |
| 29 Protein secretion | pathway | SCAMp3 | N/A |
| 30 Unfolded protein response | pathway | SHC1, ATF6 | N/A |
| 31 Reactive oxygen species pathway | pathway | NDUFS2, SBNO2 | N/A |
| 32 E2f targets | proliferation | N/A | N/A |
| 33 G2m checkpoint | proliferation | HOXC10, TLE3 | N/A |
| 34 Myc targets v1 | proliferation | N/A | N/A |
| 35 Myc targets v2 | proliferation | N/A | N/A |
| 36 p53 pathway | proliferation | GADD45A, RALGDS | N/A |
| 37 Mitotic spindle | proliferation | ARL8A, ARHGEF2 | TIAM1 |
| 38 Androgen response | signaling | N/A | N/A |
| 39 Estrogen response early | signaling | SLC39A6, MUC1, AREG, CELSR1, MYB,  BCL2, TJp3, SLC1A1, RET | TIAM1 |
| 40 Estrogen response late | signaling | AREG, MYB, BCL2, TJp3, RET, pLXNB1, NAB2 | pLAC1, TIAM1 |
| 41 IL-2 JAK STAT5 signaling | signaling | MUC1, BCL2, TNFRSF18 | SLC39A8, ETV4, TIAM1 |
| 42 KRAS signaling upregulated | signaling | N/A | ETV4 |
| 43 KRAS signaling downregulated | signaling | GAMT | N/A |
| 44 Mtorc1 signaling | signaling | N/A | N/A |
| 45 Notch signaling | signaling | ApH1A | N/A |
| 46 PI3K AKT MTOR signaling | signaling | pAK4 | TIAM1 |
| 47 Hedgehog signaling | signaling | CELSR1, LDB1, TLE3, | N/A |
| 48 TGF-beta signaling | signaling | N/A | N/A |
| 49 TNFA signaling via NFKB | signaling | MCL1, EFNA1, AREG, GADD45A, NFKB1, | N/A |
| 50 WNT beta catenin signaling | signaling | NCSTN | N/A |

Table S3. Pathway Analysis of 38 identified genes in the signature for regressing the tumor volume feature

| Category | P-value | Corrected p-value |
| --- | --- | --- |
| Glycosaminoglycan biosynthesis -keratan sulfate | 0.013982 | 0.127763 |
| Regulation of actin cytoskeleton | 0.015647 | 0.127763 |
| Terpenoid backbone biosynthesis | 0.020039 | 0.127763 |
| Glycosphingolipid biosynthesis - lacto and neolacto series | 0.023484 | 0.127763 |
| Other types of O-glycan biosynthesis | 0.027774 | 0.127763 |
| Porphyrin and chlorophyll metabolism | 0.037147 | 0.141505 |
| N-Glycan biosynthesis | 0.043066 | 0.141505 |
| Melanoma | 0.061440 | 0.159365 |
| Adherens junction | 0.063919 | 0.159365 |
| TGF-beta signaling pathway | 0.072136 | 0.159365 |

Table S4. Pathway analysis of 57 identified genes in the signature for regressing the maximum probability feature from slow-flow associated tumour subregion

| Category | P-value | Corrected p-value |
| --- | --- | --- |
| Transcriptional misregulation in cancer | 0.028136 | 0.23353 |
| Terpenoid backbone biosynthesis | 0.032421 | 0.23353 |
| Regulation of actin cytoskeleton | 0.038788 | 0.23353 |
| Regulation of autophagy | 0.057071 | 0.23353 |
| Porphyrin and chlorophyll metabolism | 0.059772 | 0.23353 |
| Bile secretion | 0.098087 | 0.23353 |
| Inositol phosphate metabolism | 0.098087 | 0.23353 |
| Melanoma | 0.098087 | 0.23353 |
| Pathways in cancer | 0.111703 | 0.23353 |
| Taste transduction | 0.113489 | 0.23353 |
