# Supplementary figures and images for: Tumour heterogeneity revealed by unsupervised decomposition of dynamic contrast-enhanced magnetic resonance imaging is associated with underlying gene expression patterns and poor survival in breast cancer patients
Source: Breast Cancer Res. 2019 Oct 17;21:112. doi: 10.1186/s13058-019-1199-8 (PMC6798414; doi:10.1186/s13058-019-1199-8)

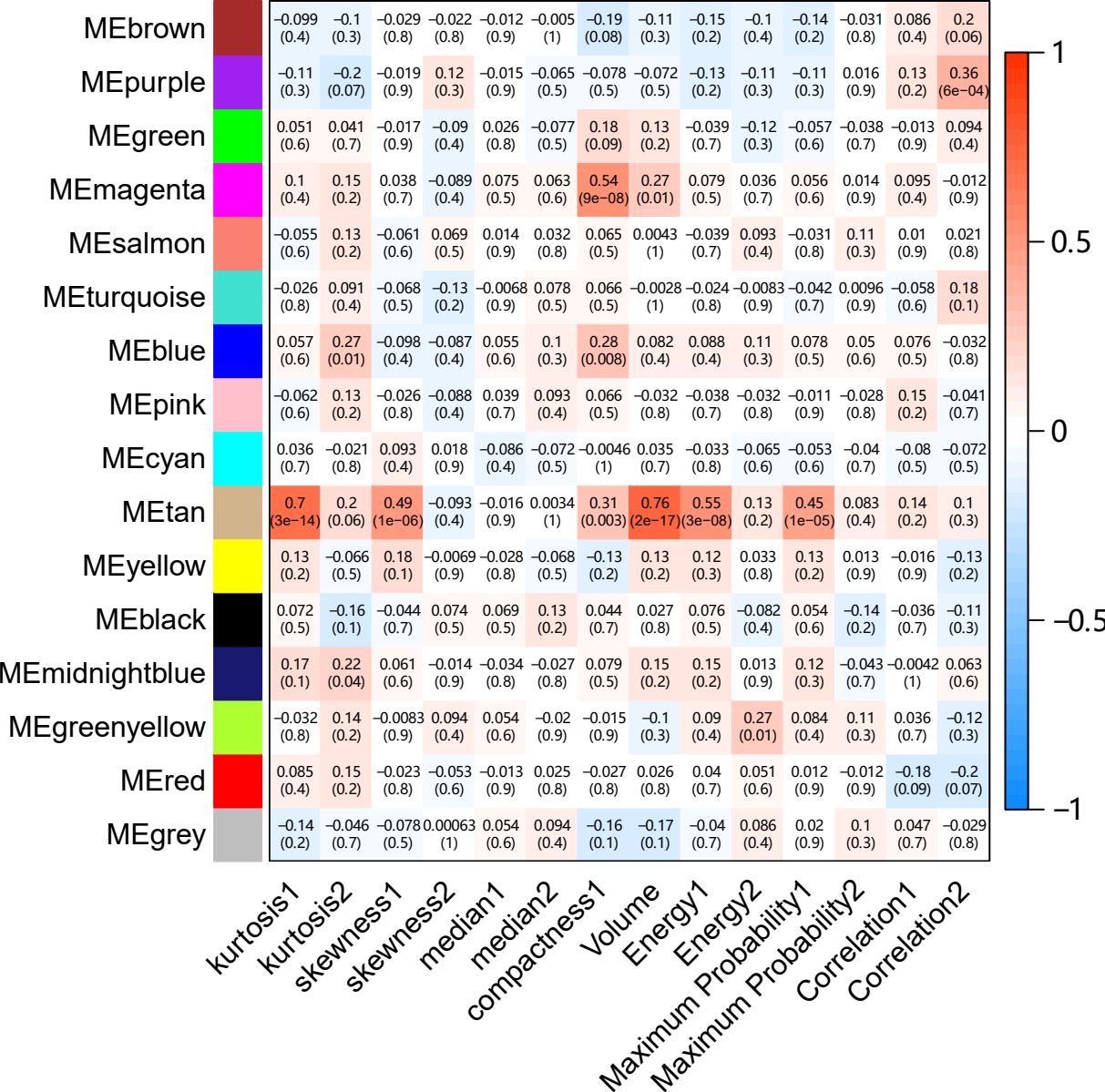

Supplement: Supplementary file 2 — Additional file 2: Figure S1. Image features from the plasma input subregion correlated with gene modules. [file 13058_2019_1199_MOESM2_ESM.pdf]

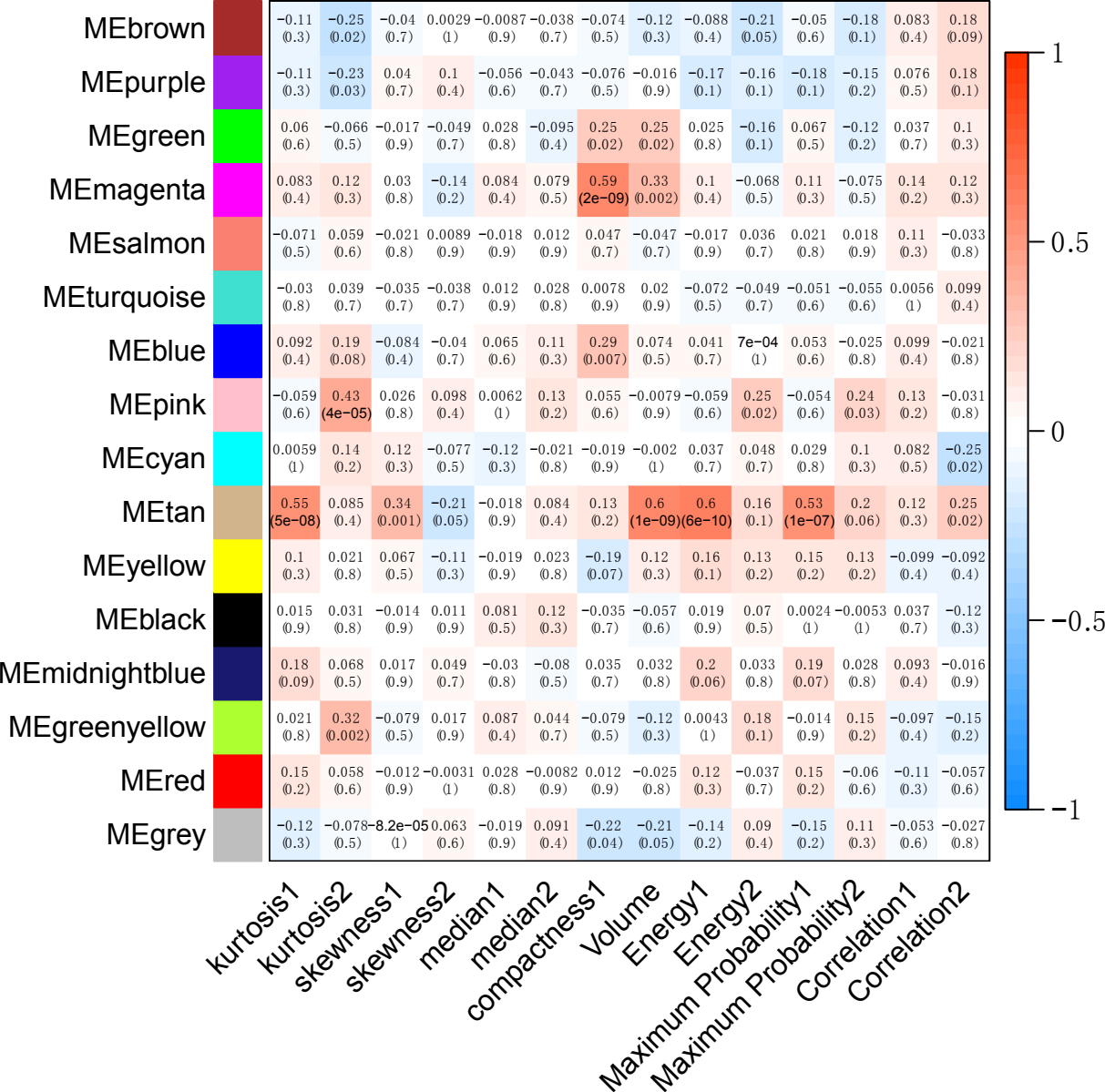

Supplement: Supplementary file 3 — Additional file 3: Figure S2. Image features from the slow-flow kinetics subregion correlated with gene modules. [file 13058_2019_1199_MOESM3_ESM.pdf]
